# Preparation of a 3D printable high-performance GelMA hydrogel loading with magnetic cobalt ferrite nanoparticles

**DOI:** 10.3389/fbioe.2023.1132192

**Published:** 2023-03-02

**Authors:** Yiwan Shi, Zhaozhen Wang, Xinting Zhou, Chengxiong Lin, Chao Chen, Botao Gao, Weikang Xu, Xiaofei Zheng, Tingting Wu, Huajun Wang

**Affiliations:** ^1^ Department of Bone and Joint Surgery and Sports Medicine Center, The First Affiliated Hospital, Jinan University, Guangzhou, China; ^2^ National Engineering Research Center for Healthcare Devices, Guangdong Key Lab of Medical Electronic Instruments and Polymer Material Products, Institute of Biological And Medical Engineering, Guangdong Academy of Sciences, Guangzhou, China; ^3^ Department of Orthopedics, School of Traditional Chinese Medicine, Southern Medical University, Guangzhou, China; ^4^ The Guangzhou Key Laboratory of Basic and Translational Research on Chronic Diseases, The First Affiliated Hospital, Jinan University, Guangzhou, China

**Keywords:** osteosarcoma, cobalt ferrite nanoparticles, GelMA, hydrogel, magnetic hyperthermia

## Abstract

Osteosarcoma remains a worldwide concern due to the poor effectiveness of available therapies in the clinic. Therefore, it is necessary to find a safe and effective therapy to realize the complete resection of osteosarcoma and reconstruction of the bone defect. Magnetic hyperthermia based on magnetic nanoparticles can kill tumor cells by raising the temperature without causing the side effects of conventional cancer treatments. This research aims to design a high-performance magnetic hydrogel composed of gelatin methacrylate and highly magnetic cobalt ferrite (CFO) nanoparticles for osteosarcoma treatment. Specifically, CFO is surface functionalized with methacrylate groups (MeCFO). The surface modified CFO has good biocompatibility and stable solution dispersion ability. Afterward, MeCFO nanoparticles are incorporated into GelMA to fabricate a three-dimensional (3D) printable MeCFO/GelMA magnetic hydrogel and then photocross-linked by UV radiation. MeCFO/GelMA hydrogel has high porosity and swelling ability, indicating that the hydrogel possesses more space and good hydrophily for cell survival. The rheological results showed that the hydrogel has shear thinning property, which is suitable as a bioprinting ink to produce desired structures by a 3D printer. Furthermore, 50 μg/mL MeCFO not only decreases the cell activity of osteosarcoma cells but also promotes the osteogenic differentiation of mBMSCs. The results of the CCK-8 assay and live/dead staining showed that MeCFO/GelMA hydrogel had good cytocompatibility. These results indicated that MeCFO/GelMA hydrogel with potential antitumor and bone reconstruction functions is a promising therapeutic strategy after osteosarcoma resection.

## 1 Introduction

Osteosarcoma is one of the most common primary bone cancers in children, adolescents, and young adults. It causes a serious threat to human health due to its strong invasiveness to local tissue, early distant metastasis, and strong postoperative obstinacy (Zhao et al., 2021). At present, the standard therapeutic strategy for osteosarcoma is the combination of surgery and adjuvant chemotherapy in the clinic (Isakoff et al., 2015). However, tumor resection is one of the most commonly used surgical treatments, which often results in extensive destruction of normal surrounding tissues and cells. Once the lesion is not completely resected, the risk of osteosarcoma recurrence is high (Kim et al., 2010). On the other hand, the drug resistance of osteosarcoma and the organ damage side effects of chemotherapeutic drugs (such as cisplatin, doxorubicin, and ifosfamide) impair the tumor therapeutic effect (Rathore and Van Tine, 2021). Therefore, most patients with osteosarcoma have a poor prognosis, which presents a challenging treatment dilemma.

Recently, hyperthermia therapy has attracted more and more attention in cancer treatment due to the thermal sensitivity of tumor cells and negligible side effects (Kang et al., 2020). Compared to photothermal therapy, high-intensity focused ultrasound and radiofrequency ablation, magnetic hyperthermia therapy rapidly reaches temperature elevation in tumor tissues by the magnetic thermal conversion of magnetic nanoparticles under an alternating magnetic field (Beik et al., 2016; Liu et al., 2020). And thus, moderate temperature (40°C–45°C) can kill or induce tumor cell apoptosis without damaging normal cells, which provides a suitable strategy for the treatment of bone cancer (Jose et al., 2020). In the past few decades, the synthesis of magnetic nanoparticles has been constantly explored and developed, including magnetic metal nanoparticles, magnetic metal alloy, magnetic metal oxide nanoparticles, and multifunctional magnetic nanoparticles (Wu et al., 2016). Among the different magnetic materials, iron oxide nanoparticles have been widely studied due to their good biocompatibility (Elahi and Rizwan, 2021). In addition to maintaining the advantages of iron oxide nanoparticles, cobalt ferrite (CoFe_2_O_4_, CFO) nanoparticles exhibit better magnetic behavior, including saturation magnetization, coercivity, and magnetocrystalline anisotropy (Srinivasan et al., 2018). Because of these good properties, they are widely used in biomedicine, such as drug delivery systems (Wang et al., 2018), medical imaging (Alazmi et al., 2019), and cancer treatment (Hatamie et al., 2021). For biomedical applications, magnetic nanoparticles should be non-toxic materials or coated with substances that assure their stability and biocompatibility. Nanoparticles tend to form agglomerates due to attractive van der Waals forces (Chuan Lim and Feng, 2012). To obtain stable nanoparticles, it is necessary to coat magnetic nanoparticles with a protective layer, such as polymer and carbon (Ahangaran and Navarchian, 2020).

Besides, the degradation of CFO nanoparticles by lysozyme releases Co^2+^ *in vivo* (Volatron et al., 2017). As a kind of heavy metal, Co^2+^ acts as an anti-tumor agent comparable to chemotherapy drugs (Balakrishnan et al., 2020). But the application of high concentrations of CFO nanoparticles is cytotoxic to normal cells in surrounding tissues, which limits its widespread use in the medical field. It has been reported that the CFO modified by surface coating reduced its cytotoxicity (Di Guglielmo et al., 2010). In this study, CFO was surface-grafted by methacrylate groups (labeled as MeCFO). The Methacrylate group is a common group of silane coupling agents. It can also reduce nanoparticle agglomeration and improve cytocompatibility (Nguyen and Shim, 2012; Mamat et al., 2019). Compared with other silane coupling agents [3-(triethoxysilyl) propyl methacrylate and triethoxyvinylsilane], 3-(trimethoxysilyl)-propyl methacrylate (TMSPMA) not only has better stability but also higher modification efficiency (Atila Dinçer et al., 2014).

To repair bone defects caused by osteosarcoma resection, the subsequent bone reconstruction by implanted biomaterials is as significant as the killing of tumor cells. The selection of appropriate biomaterials to fill the defect area is also a crucial step in the treatment of bone tumors (Ma et al., 2022). Hydrogel is a crosslinked polymer system with physical and chemical properties similar to natural tissue. It is a good candidate for an extracellular matrix with the advantages of excellent biocompatibility, biodegradability, and high water-retention ability (Chen et al., 2022a). As a result, it can be used as an excellent storage medium for hyperthermia nanoparticles and an ideal scaffold material for tissue engineering. Gelatin methacryloyl (GelMA) is a derivative of gelatin, which retains the cell-binding sequence Arg-Gly-Asp (RGD) and matrix metalloproteinase (MMP)-sensitive degradation motifs (Monteiro et al., 2018). Therefore, it is beneficial to cell adhesion and has good biocompatibility. GelMA can be cross-linked to form a hydrogel under ultraviolet (UV) or blue light radiation (Zu et al., 2021). Thus, it is easier to be made it into the desired 3D structure for regulating cell behaviors. As an ideal biomaterial, it has been widely used in various tissue engineering fields. However, the mechanical properties of GelMA are poor and need to be strengthened.

The combination of CFO and GelMA is expected to improve the mechanical strength of GelMA hydrogel and the biocompatibility of CFO. Therefore, this work aims to prepare methacrylate group coated CFO nanoparticles by surface functionalization with TMSPMA. Moreover, the prepared MeCFO nanoparticles were added to GelMA hydrogel to obtain a high-performance magnetic hydrogel for magnetic hyperthermia of osteosarcoma. The physicochemical feature and cytocompatibility *in vitro* of the composite hydrogel are evaluated experimentally, aiming to provide a theoretical basis for broadening its biomedical application in osteosarcoma treatment.

## 2 Materials and methods

### 2.1 Materials

CFO nanoparticles, 3-(trimethoxysilyl)-propyl methacrylate (TMSPMA) and 2-hydroxy-4-(2-hydroxyethoxy)-2-methylpropiophenone (Irgacure-2959, I2959) were purchased from Macklin (Shanghai, China). GelMA was purchased from Engineering For Life (Suzhou, China). All the reagents for cell cultures were purchased from VivaCell (Shanghai, China). Other reagents for cell experiments were purchased from Beyotime Biotechnology (Shanghai, China) unless otherwise mentioned.

### 2.2 Synthesis of MeCFO nanoparticles

MeCFO was synthesized from a slightly modified procedure reported previously (Cha et al., 2014). CFO nanoparticles were conjugated with methacrylate functional groups by reacting with TMSPMA. Briefly, CFO nanoparticles were evenly dispersed in anhydrous ethanol by ultrasound for 20 min. The TMSPMA solution was slowly added to CFO suspension with a ratio of 50 μL per mg CFO. The reaction mixture was stirred mechanically for 12 h at 50°C. And it was dialyzed in anhydrous ethanol for 3 days. Finally, MeCFO was obtained by vacuum drying and kept in a dark place.

### 2.3 Fabrication of MeCFO/GelMA hydrogel

The hydrogel was prepared by crosslinking under ultra-violet (UV) (405 nm) light with I2959 as the photoinitiator. It mainly consisted of 5% GelMA doped with different concentrations of MeCFO (Table 1). Briefly, GelMA was dissolved in phosphate buffered saline (PBS) at 60°C. The photoinitiator I2959 was completely dissolved in PBS at 40°C in dark. MeCFO was sonicated and dispersed in PBS for 2 h. Then GelMA was added to the MeCFO dispersion and thoroughly mixed. At last, the I2959 solution was mixed into the above system with a final concentration of 0.1%. The solution was then transferred to a polymethyl methacrylate mold and formed by UV light for 10 min.

**TABLE 1:** Nomenclature and composition of MeCFO/GelMA hydrogels.

Hydrogel label	GelMA (mg/mL)	MeCFO (mg/mL)	I2959 (mg/mL)
0%MeCFO/GelMA	50	0	1
0.01%MeCFO/GelMA	50	0.005	1
0.05%MeCFO/GelMA	50	0.025	1
0.1%MeCFO/GelMA	50	0.05	1

### 2.4 Characterization of MeCFO nanoparticles and MeCFO/GelMA hydrogel

#### 2.4.1 Morphology and composition

The surface morphology of MeCFO nanoparticles was observed by transmission electron microscopy (TEM, JEOL JEM 2100). And their elemental analyses were qualitatively characterized by an energy dispersive X-ray spectrometer (EDX, Oxford X-Max TEM). The spectrophotometer (Thermo Scientific iN10) was used to get Fourier-transform infrared (FTIR) spectra of magnetic nanoparticles in the range of 400–4,000 cm^-1^ using KBr pellets to confirm surface functionalization of CFO nanoparticles. The element chemical states were presented with X-ray photoelectron spectroscopy (XPS, Thermo Scientific K-Alpha). The XPS spectrum for CFO and MeCFO were recorded to evaluate the covalent bonding of the silane coupling agent. The spectrum were recorded on a K-Alpha instrument (Thermo Scientific), using a monochromated Al Kα source (1,486.6 eV) at a pressure of 5 × 10^−7^ mbar. The thermogravimetric measurements of magnetic nanoparticles were made in the temperature range of 30–800°C under nitrogen gas (heat rate 10°C/min) using a thermogravimetric analyzer (TGA, Discovery TGA 550). The magnetic properties of MeCFO nanoparticles were analyzed by a vibrating sample magnetometer (VSM, LakeShore 7404) at 25°C from −20 kOe to 20 kOe. Meanwhile, we also calculated the temperature field using COMSOL Multiphysics 5.6 software according to previous studies (Zhang et al., 2020; Hossain and Hossain, 2022). The release of Fe^3+^, Co^2+^, and Si^4+^ from magnetic nanoparticles had been detected by inductively coupled optical emission spectroscopy (ICP-OES, Agilent 5110). To determine the ion release of CFO and MeCFO, 10 mg nanoparticles were immersed in 1 mL Tris-HCl solution (0.01 M, pH = 7.4). The supernatants at different time points were separated by centrifugation and measured by ICP-OES.

For MeCFO/GelMA hydrogel, the surface chemistry was determined with a Fourier transform infrared spectrometer (Bruker Tensor 27) in the region from 4,000 to 500 cm^–1^. The microstructure of hydrogel was observed by scanning electron microscope (SEM, Phenom).

#### 2.4.2 Mechanical test

The MeCFO/GelMA hydrogel solution was injected into the mold and solidified into a cylinder shape (a height of 5 mm and a diameter of 6 mm) by UV light for the compression test. The compression test was performed by a compress test machine with a cross speed of 2 mm*min^−1^. The overall strain-stress curve of the material was nonlinear, but the initial region was approximately linear. Therefore, strains in the range of 5%–15% were selected for analysis to determine the compression modulus. For each test, three samples were used and their average was calculated. The stress (σ) at 5%–15% strain was calculated according to Eq. 1:

$$\sigma =\frac{F}{S}\times 100\%$$ (1)

#### 2.4.3 Rheological characterization

The viscoelastic properties of the hydrogels were evaluated by using a parallel plate rotor (25 mm diameter) of the Anton Paar MCR302 Rheometer. Different concentrations of MeCFO/GelMA hydrogel precursor solutions were transferred to the rheometer sample stage. The rheometer was set at 10°C. The viscoelastic tests include flow sweep scanning, amplitude scanning, frequency scanning, and time scanning. For flow sweep scanning, the shear rate was set as 0.1–100 S^−1^. The variation trend of sample viscosity with a shear rate was observed. In amplitude scanning, shear strain ranges from 0.01% to 100%. It could be observed that the storage modulus G′ and the loss modulus G″ of the sample vary with the amplitude. For frequency scanning, the strain value on the operating interface was selected as “constant value”. The shear strain was 0.5%. The angular frequency is 0.1–100 rad/s. The time interval was determined by the device. The sample modulus G′ and G″ was recorded as a function of frequency. For time scanning, the scanning time was set as 5 min. The modulus G′ and G″ of samples were traced with time. All rheological measurements were made in triplicate.

#### 2.4.4 Determination of porosity

The porosity was determined by an ethanol displacement method. Specifically, the weight of dry hydrogel before soaking was denoted as W_1._ The volume of the sample was measured and labeled as V. Then the sample was immersed in ethanol. The hydrogel was vacuumed with a vacuum pump until it stopped bubbling. The sample was taken out and weighed (W_2_). The porosity was calculated according to Eq. 2 and displayed as mean ± standard deviation (*n* = 3).

$$Porosity\;\left(P\right)=\left(W_{2}–W_{1}\right)/\;\left(\rho V\right)\times 100\%$$ (2)

#### 2.4.5 Swelling study

To evaluate the water absorption capacity of MeCFO/GelMA hydrogel, swelling studies were performed as follows. Briefly, the freeze-dried hydrogel was weighed and marked as the initial weight W_3_. Then they were immersed in PBS solution, and placed on a 72 rpm shaker at 37°C to swell. At scheduled time intervals, samples were taken out. Meanwhile, the water on the surface of the samples was washed off with absorbent paper and recorded the weight (W_4_). After each measurement, the hydrogel was immersed in PBS for further swelling, The swelling ratio (SR) was determined according to Eq. 3. Three samples from each group were selected for the swelling test.

$$SR=\left(W_{4}-W_{3}\right)/\;W_{3}\times 100\%$$ (3)

### 2.5 Preparation of MeCFO/GelMA hydrogel bioink and 3D printing

To increase the GelMA viscosity and make GelMA undergo reversible physical cross-linking, the prepared MeCFO/GelMA solution was placed at 4°C for 20 min before printing. Then the printer cartridge was loaded onto the biological 3D printer with the low-temperature mode. The platform temperature was controlled at 4°C. The temperature of the print nozzle was 24°C. The pressure of the printing extrusion was controlled to be about 200 kPa. The moving speed of the dispensing unit was set to 200 mm/s, and the printing height and spacing were respectively 0.23 mm and 1 mm. After setting the parameters, we adjusted the size and generation path of the 3D model as required. The prepared MeCFO/GelMA solution was easily extruded from the needle due to shear thinning. When it dropped onto the printing platform (4°C), it quickly restored viscosity. To solidify the printed scaffolds, UV light curing was carried out synchronously in the printing process. Finally, the printed pre-scaffold was irradiated with UV light for 10 min.

### 2.6 Cell experiments

#### 2.6.1 Cell culture

Bone mesenchymal stem cells (BMSCs) were taken from the bone marrow of Sprague Dawley (SD) rats. Human osteosarcoma (SAOS-2) cells were purchased from the company (Zhong Qiao Xin Zhou, China). They were used to evaluate cell proliferation and cell viability *in vitro*. The culture medium of BMSCs or Saos-2 cells was composed of high glucose DMEM, 10% FBS, and 1% penicillin-streptomycin. And they were cultured at 37°C with 5% carbon dioxide. The medium was changed every twice day. When the cells grow full and digested prepare a cell suspension with a density of 10^6^ cells/mL for the following experiments. All powder samples were fully sterilized by damp-heat sterilization at 120°C and dried. For the hydrogel samples, they were prepared under a sterile condition with sterilized raw materials.

#### 2.6.2 Cytocompatibility assay of BMSCs

The cytocompatibility assay was accomplished by BMSCs *in vitro*. We used the CCK-8 method to assess the cytocompatibility of magnetic nanoparticles and hydrogels. Briefly, sterilized CFO or MeCFO was immersed in DMEM medium to obtain material extract (200 μg/mL). The material extracts were diluted into different gradients according to the experimental design. The BMSCs were inoculated in 96-well plates at a density of 4×10^3^ cells/well. After the next day, it was changed into the material extract and co-cultured for 1 and 4 days. The cytocompatibility was evaluated by CCK-8 assay at the time points. CCK-8 solution and DMEM medium were mixed evenly at a volume ratio of 1:10 and then added. After incubating for 90 min, the cytocompatibility was evaluated by measuring the optical density of CCK-8 solution at 450 nm using a microplate reader (Tecan).

For the cytocompatibility of hydrogels with different solid contents of MeCFO, 20 µL MeCFO/GelMA hydrogel was placed into 96-well plates and was formed *in situ* by UV light. Next, 3×10^3^ cells were seeded onto the scaffolds and incubated for 4 days as mentioned above. Every other day, the medium was replaced with a fresh medium to maintain cell activity.

The viability of BMSCs on the hydrogels was determined according to the instructions of the live-dead cell staining kit. Briefly, 3×10^3^ BMSCs were seeded onto the scaffolds and incubated for 4 days. After 4 days, the cells were washed 2 times with PBS and treated with ethylenediamine homodimer-1 (0.5 μM) and calcein AM (0.25 μM) at 37°C for 20 min. Cells on the hydrogel were observed using an inverted fluorescence microscope (Leica DMi8). The excitation filter was set at 488 nm to observe live (green) cells and dead (red) cells at 561 nm.

#### 2.6.3 Cytotoxicity assay of Saos-2 with CFO or MeCFO

The Saos-2 cells were used to verify the antitumor effect of MeCFO. According to the above experimental exploration, an appropriate concentration of the material extract was selected and co-cultured with Saos-2 for 1 and 4 days. CCK-8 experiment was carried out after reaching the predetermined time point.

#### 2.6.4 Alizarin red staining (ARS)

Alizarin red is a dye that selectively binds calcium salts. Mineralization of materials can be evaluated by alizarin red staining. For the osteogenic differentiation assay, BMSCs were cultured in the cell culture medium containing osteogenic supplements, including vitamin C, dexamethasone, and sodium glycerophosphate. Then 1×10^4^ BMSCs were co-cultured with the material extract. The osteogenic differentiation medium containing the material extract was replaced every 2 days for 7 days. After 7 days, every well was washed with PBS and fixed in 4% paraformaldehyde for 2 h. Finally, they were then stained with ARS solution for 30 min. After the excess dye is washed off, the images are obtained from each well by an inverted microscope (Leica DMi1).

#### 2.6.5 Reverse transcription-quantitative polymerase chain reaction (RT-qPCR)

The expression of osteogenic genes of magnetic nanoparticles was quantitatively determined by RT-qPCR. In this study, BMSCs were co-cultured with material extracts containing osteogenic differentiation medium for 7 days. To analyze RNA expression, each well was washed with PBS and fully cleaved with lysate. RNA of the samples was extracted by Hipure Total RNA Micro Kit (Magen) according to the kit instructions. RNA concentration was detected by a microspectrophotometer (Thermo scientific). We then reverse-transcribed the extracted RNA into cDNA using the Transcript First Strand cDNA Synthesis Kit (Roche). According to the manufacturer’s protocol, relevant osteogenic differentiation gene primers and SYBR green reagent were added for RT-qPCR. The instrument of RT-qPCR detection was performed using StepOnePlus real-time PCR system (Thermo Fisher Scientific). Osteogenic gene primers were listed in Table 2.

**TABLE 2:** The list of primer names and sequences (5′-3′).

Gene names	Primer sequences (5′-3′)
Glyceraldehyde-3-p dehydrogenase (GAPDH)	F: 5′-AGC​CCA​GAA​CAT​CAT​CCC​TG-3′
	R: 5′-CAC​CAC​CTT​CTT​GAT​GTC​ATC-3′
Bone morphogenic protein-2 (BMP-2)	F: 5′-GAA​GCC​AGG​TGT​CTC​CAA​GAG-3′
	R: 5′-GTG​GAT​GTC​CTT​TAC​CGT​CGT-3′
Vascular endothelial growth factor (VEGF)	F: 5′-AGG​CTG​CAC​CCA​CGA​CAG​AA-3′
	R: 5′-CTT​TGG​TCT​GCA​TTC​ACA​TC-3′

### 2.7 Statistical analysis

All experimental data were analyzed by Origin 2021 software (OriginLab Corporation, United States). Their results were presented as a mean ± standard deviation. Statistical analysis was performed using the two-tailed Student’s *t*-test between different groups. One-way analysis of Variance (ANOVA) was used to compare and analyze multiple data sets. The level of significant statistical differences was set as a *p*-value less than 0.05.

## 3 Results and discussion

### 3.1 Synthesis and characterization of MeCFO

**FIGURE 1:**
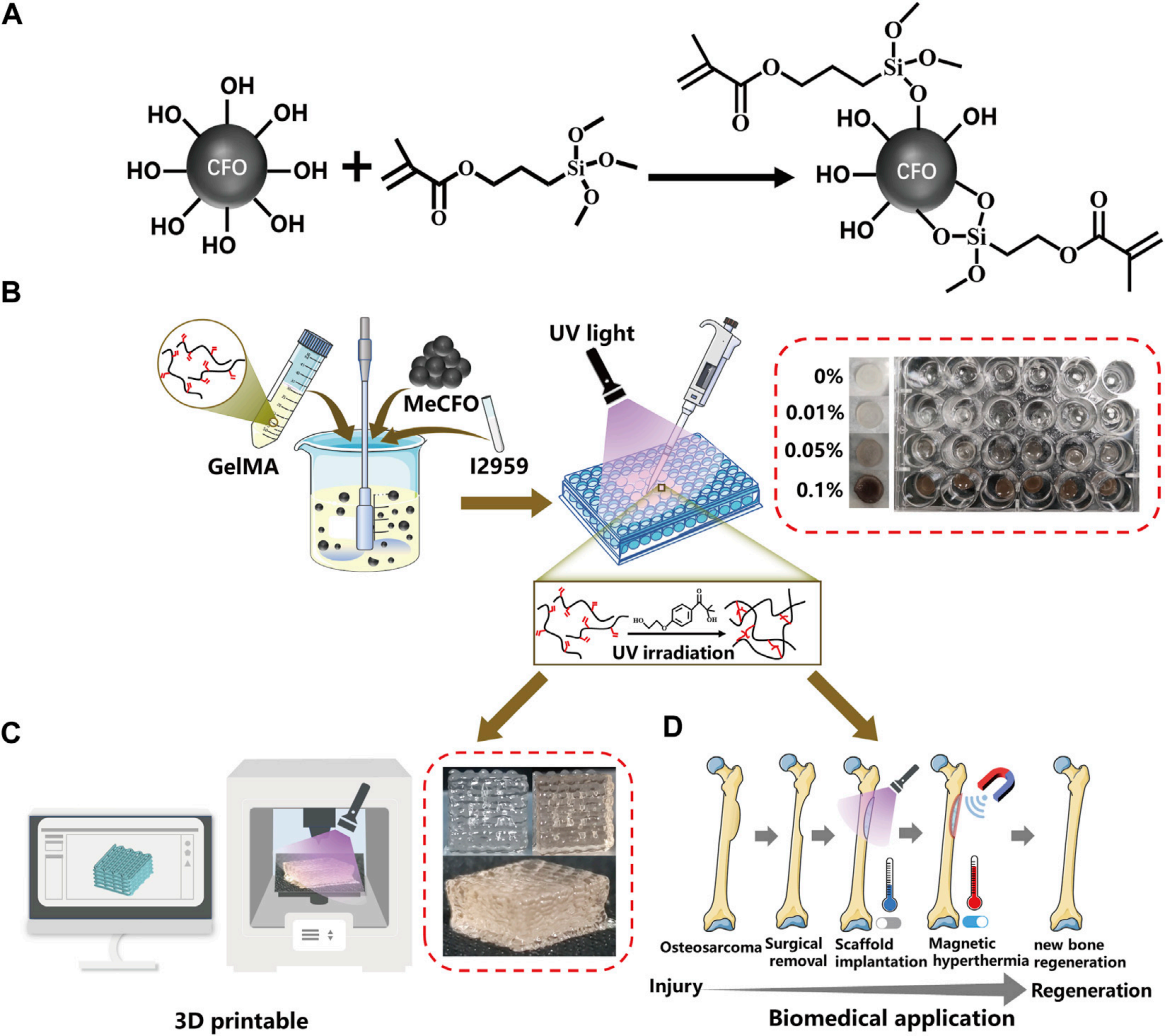
Schematic diagram of synthetic routes of MeCFO/GelMA hydrogels. **(A)** synthesis of MeCFO; **(B)** preparation of MeCFO/GelMA hydrogels; **(C)** 3D printing; **(D)** Magnetic hyperthermia application of MeCFO/GelMA hydrogels.

**FIGURE 2:**
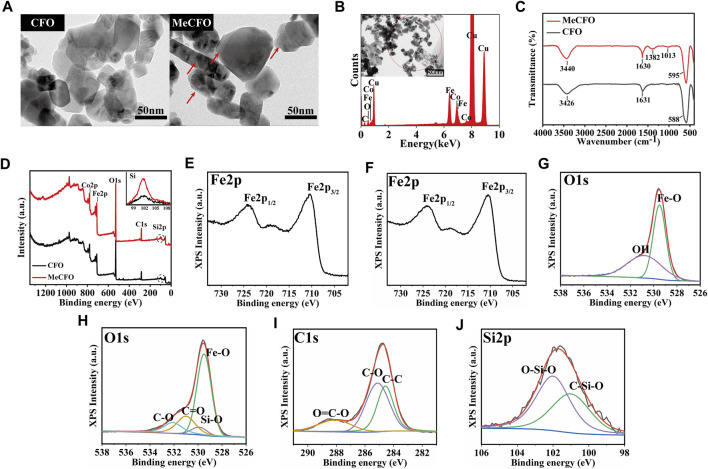
The morphology and composition analysis of the CFO and MeCFO. **(A)** the TEM images of CFO and MeCFO; **(B)** the EDX spectrum of MeCFO; **(C)** the FTIR spectrum of CFO and MeCFO; **(D)** the XPS spectrum of CFO and MeCFO; **(E)** XPS spectra of Fe2p of CFO; **(F)** XPS spectra of Fe2p of MeCFO; **(G)** XPS spectra of O1s of CFO; **(H)** XPS spectra of O1s of MeCFO; **(I)** XPS spectra of C1s of MeCFO; **(J)** XPS spectra of Si2p of MeCFO.

The surface of CFO nanoparticles in this study was chemically modified to reduce their agglomeration (Atila Dinçer et al., 2014; Sundar et al., 2014). For this purpose, methacrylate groups were conjugated on the surface of CFO by reaction with TMSPMA to obtain MeCFO (Figure 1A). TEM images illustrated the morphology and size of MeCFO. As shown in Figure 2A, CFO and MeCFO were both polygonal in shape with an average diameter of around 20–100 nm. Compared with CFO, there was a slight difference in the morphology of the magnetic nanoparticles after surface modification. The modified CFO nanoparticles were thicker as they were coated with a thin TMSPMA layer. Moreover, CFO nanoparticles were aggregated and overlapped while MeCFO nanoparticles were dispersed with little overlap. This indicates that TMSPMA enhanced the dispersion of magnetic nanoparticles, which was conducive to the dispersion of nanoparticles in the substrate biomaterial (Atila Dinçer et al., 2014; Tanasa et al., 2019).

Figure 2B showed the main elemental composition of CFO and MeCFO by EDX spectrum. The presence of the elements, C, Fe, Co, and O, were appeared in the MeCFO. But Si in MeCFO was not found by EDX spectrum analysis due to the low content of Si. FTIR spectra (Figure 2C) confirm the successful synthesis of MeCFO by identifying the functional group of its organic molecules. The FTIR spectrum CFO and MeCFO were found characteristic peaks at 580–590 cm^-1^ due to the stretching vibration of the Fe-O bond (Bohara et al., 2014). The absorption bands around 3,400 cm^-1^ and 1,631 cm^-1^ are attributed to the bending and stretching vibrations of water molecules (Penchal Reddy et al., 2015). The absorption bands near 1,382 cm^-1^ and 1,013 cm^-1^ respectively corresponded to the vibration of the Si-C bond and vibration of the Si-O bond from TMSPMA (Cha et al., 2014; Koohestani and Sadjadi, 2021). The hydroxyl functional group of CFO nanoparticles was transformed into the methacrylate group by silanization. Because of the existence of C=O bonds in TMSPMA, the absorption peak of MeCFO near 1,630 cm^-1^ was significantly stronger than that of CFO.

For further surface composition analysis of CFO and MeCFO, XPS detection was performed (Figures 2D–J). XPS spectrum can identify the Si in the MeCFO sample compared to CFO samples (Figure 2D). The XPS data of CFO nanoparticles could not be identified as silicon by Thermo Avantage v5.948 software. The peak of MeCFO nanoparticles’ binding energy at 285.08 eV was attributed to C1s, and the weak peak at 102.08 eV is related to the presence of Si2p, which further proved that TMSPMA was coated on the surface of CFO nanoparticles (Tan et al., 2013). The high-resolution Fe2p, O1s, and C1s XPS spectra were also investigated for an in-depth analysis of the MeCFO composition. In the Fe2p spectrum of CFO and MeCFO (Figures 2E, F), two obvious peaks were observed at 724.3 and 710.5 eV, ascribed to Fe2p _1/2_ and Fe2p _3/2_ of CFO, respectively (Rodewald et al., 2019). Figure 2G highlighted that the O1s peak of CFO nanoparticles had two deconvolution peaks. The first peak was concentrated at 529.5 eV, which could be attributed to Fe-O (Márquez et al., 2012). The second peak was concentrated at 530.8 eV, possibly corresponding to hydroxyl bonding within the magnetic nanoparticles (Tanasa et al., 2019). Furthermore, as shown in Figure 2H, four different kinds of O1s peaks were observed in the XPS spectra of MeCFO at 529.5, 529.9, 531, and 532.1 eV. They are assigned to Fe-O, Si-O, C=O, and C-O, respectively (Xiong et al., 2016). In the C1s spectrum of MeCFO (Figure 2I) four types of C1s peaks correspond to the following functional groups: C-C (284.5 eV), C-O (284.9 eV), C-N (285.5 eV) and O=C-O (288.1 eV), respectively (Shi et al., 2016). As seen in Figure 2J, the Si2p curve simulates two characteristic signals of 101.9 eV (C-Si-O) and 102.5 eV (O-Si-O) (Yan et al., 2022). These results further prove there are methacrylate groups on the surface of the CFO.

**FIGURE 3:**
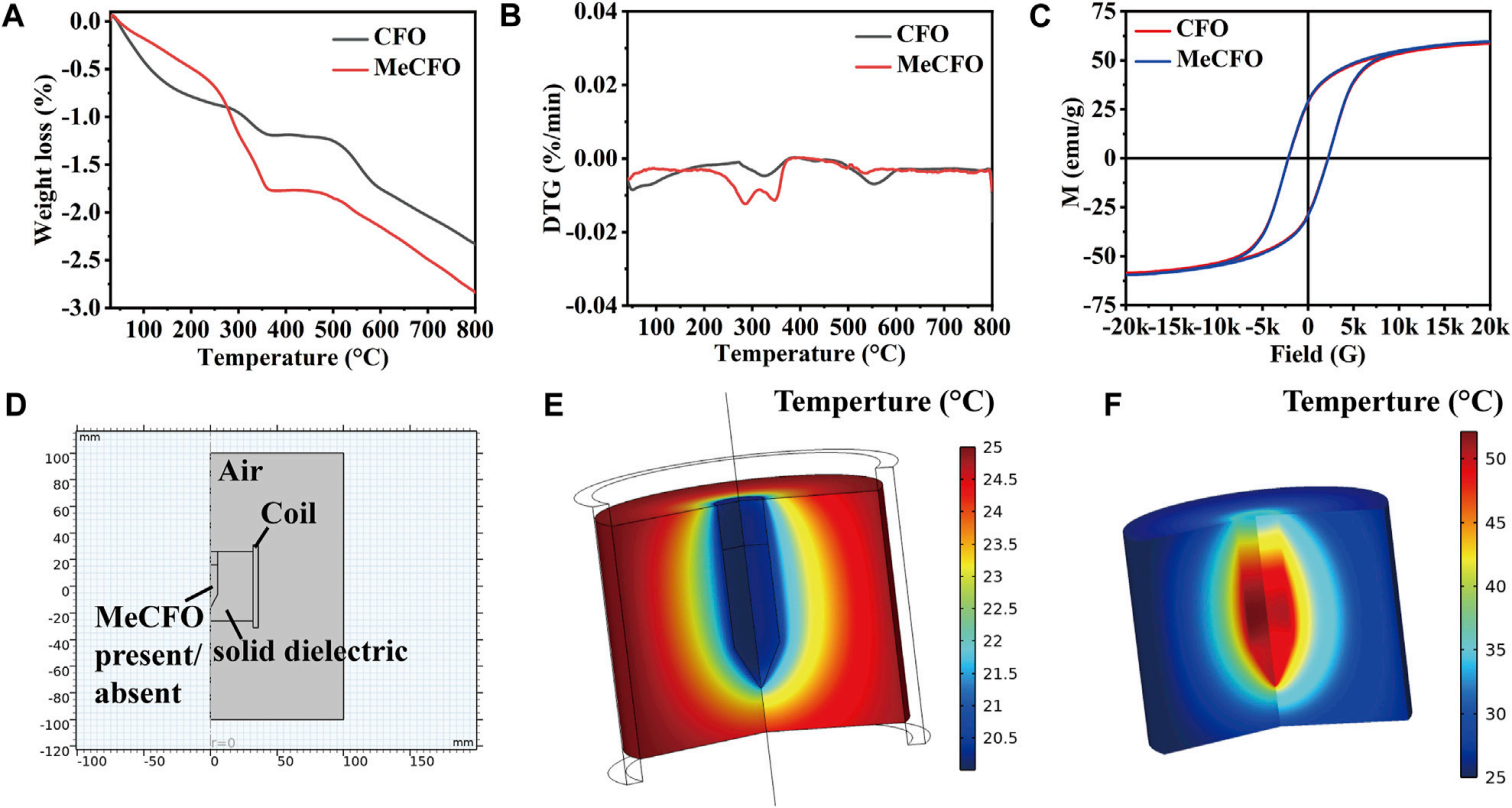
The characteristics of CFO and MeCFO. **(A)** the TG diagrams of CFO and MeCFO; **(B)** the DTG diagrams of CFO and MeCFO; **(C)** the hysteresis loop curve of CFO and MeCFO; **(D)** geometry construction of magnetothermal simulation with Comsol software; **(E)** temperature distribution without MeCFO; **(F)** temperature distribution with MeCFO.

The thermal behavior of CFO nanoparticles before and after functional modification is shown in Figures 3A, B. Figure 3A displayed that the weight of CFO and MeCFO decreased with the increase in temperature. There was a significant weight loss within 360°C. It could be the evaporation of water and pyrolysis of the unstable hydroxyl group from the magnetic nanoparticles (Zhao et al., 2012; Alam et al., 2017). The weight loss rate of MeCFO was higher than that of CFO, which is related to the continuous loss of methacrylate groups (Duong et al., 2017). Their decline rates tended to be the same above 360°C. When the temperature rose to 800°C, the weight loss ratios of CFO and MeCFO were respectively 2.337% and 2.839% (Table 3). Thermogravimetric results also demonstrated that TMSPMA was successfully grafted on the surface of CFO nanoparticles. The derivative thermogravimetric (DTG) curve of CFO and MeCFO were shown in Figure 3B. The maximum degradation temperature of CFO and MeCFO respectively were 323.57°C and 345.92°C, which is consistent with the literature description (Nguyen et al., 2020). The intensity and area of the CFO degradation peak were greater than those of the MeCFO. This also indicated that the methacrylate groups on MeCFO were decomposed by heat. Figure 3C showed the hysteresis loops of CFO and MeCFO. The saturation magnetization (Ms) values for the CFO and MeCFO samples were 58.52 and 59.56 emu/g, respectively. The coating did not significantly change the magnetization. In addition, the coercivity (Hc) of CFO and MeCFO samples were 2.17 and 2.19 KOe. According to the literature, an increase in the Hc value of MeCFO may be attributed to its increased diameter as a result of surface functionalization (Almasi Kashi et al., 2020). The coating effect of TMSPMA on CFO is close to the results reported in the references (Atila Dinçer et al., 2014; Tanasa et al., 2019; Agnihotri et al., 2022). VSM measurements show that surface modification does not cause significant changes in magnetic properties. To simulate the magnetothermal effect of MeCFO, magnetic fluid was simulated by finite element method. The temperature distribution diagrams of magnetic fluid were obtained by COMSOL Multiphysics 5.6 software (Figures 3D–F). As shown in Figure 3D, we first preset our geometric model using the software. With the condition of magnetic field, the temperature of blank group could be calculated and analyzed to be concentrated on the coil surface. At the same time, the temperature did not rise significantly (Figure 3E). Different from the blank group, the temperature distribution of MeCFO group was diffused outward by MeCFO. Moreover, the temperature of magnetic fluid reached 40°C–45°C, which could help MeCFO nanoparticles kill tumor cells (Hervault et al., 2016). The temperature of magnetic nanoparticles can be controlled by changing the concentration, intensity, and time of the magnetic field (Darwish et al., 2019). This will be conducive to the promotion of MeCFO antitumor therapy. The ion concentrations released by CFO and MeCFO nanoparticles at 1 and 4 days are shown in Table 4. It could be found that the release of Co^2+^ from the MeCFO is significantly less than that of the CFO. The Fe^2+^ of the MeCFO is significantly less than that of the CFO. Because the released Si^4+^ is too little, no change can be detected by ICP-OES within 4 days when compared with the soaking solution with Si-containing impurity (not shown). In conclusion, MeCFO nanoparticles are expected to be promising in magnetic hyperthermia.

**TABLE 3:** Thermal parameters of CFO and MeCFO.

Sample	Weight loss (%)	Maximum degradation temperature (°C)
CFO	2.337	323.57
MeCFO	2.839	345.92

**TABLE 4:** Release of elemental Fe and Co from CFO or MeCFO at different times.

Sample group	Release time (day)	Concentration (mg/L)	
		Fe	Co
CFO	1	0.0271 ± 0.0002	0.0066 ± 0.0002
MeCFO	1	0.0189 ± 0.0005	0.0064 ± 0.0004
CFO	4	0.0357 ± 0.0001	0.0058 ± 0.0001
MeCFO	4	0.0372 ± 0.0002	0.0046 ± 0.0002

### 3.2 Fabrication and characterization of MeCFO/GelMA hydrogels

**FIGURE 4:**
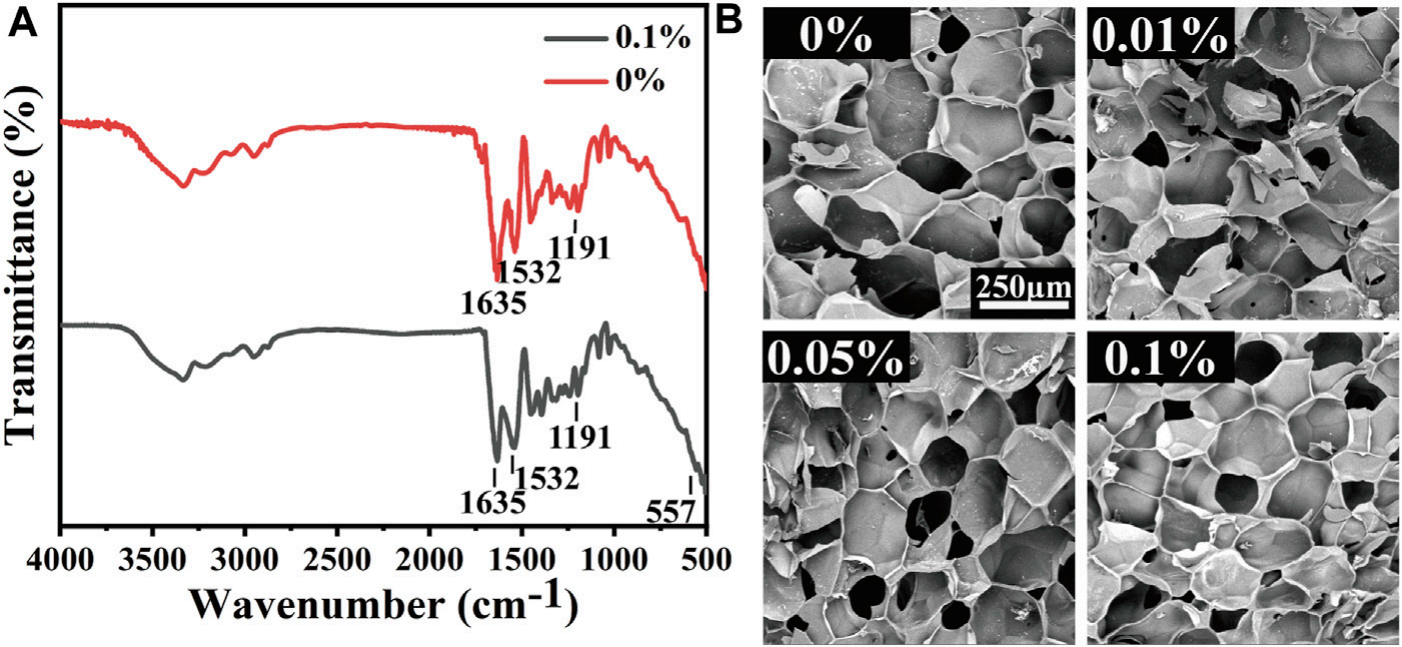
FTIR spectrum **(A)** and structure **(B)** of MeCFO/GelMA hydrogels.

The synthetic route of MeCFO/GelMA hydrogel is shown in Figure 1B. CFO nanoparticles were incorporated into GelMA solution and formed by UV light photocrosslinking. FTIR spectrogram (Figure 4A) of the composite hydrogels showed the characteristic peaks of GelMA significantly observed at 1,635, 1,532, and 1,191 cm^−1^ were respectively related to the −C=O (amide Ⅰ), N-H (amide Ⅱ), and C-O-C bonds (Rahimi Mamaghani et al., 2018; Bansal et al., 2022). An absorption band around 557 cm^−1^ appeared in the spectrum of MeCFO/GelMA hydrogel was ascribed to the MeCFO nanoparticles (Supriya et al., 2019). The cross-section microstructure of MeCFO/GelMA hydrogel was studied by SEM. The microstructure of MeCFO/GelMA hydrogel with different proportions of MeCFO is indicated in Figure 4B. All MeCFO/GelMA hydrogels exhibited a porous microstructure due to the process of freeze drying. With the increase in MeCFO content, the pore size of MeCFO/GelMA hydrogel gradually decreased. This is because the presence of MeCFO fills the porous microstructure and the number of holes in the hydrogel (Chen et al., 2022b).

### 3.3 Mechanical properties of MeCFO/GelMA hydrogel

**FIGURE 5:**
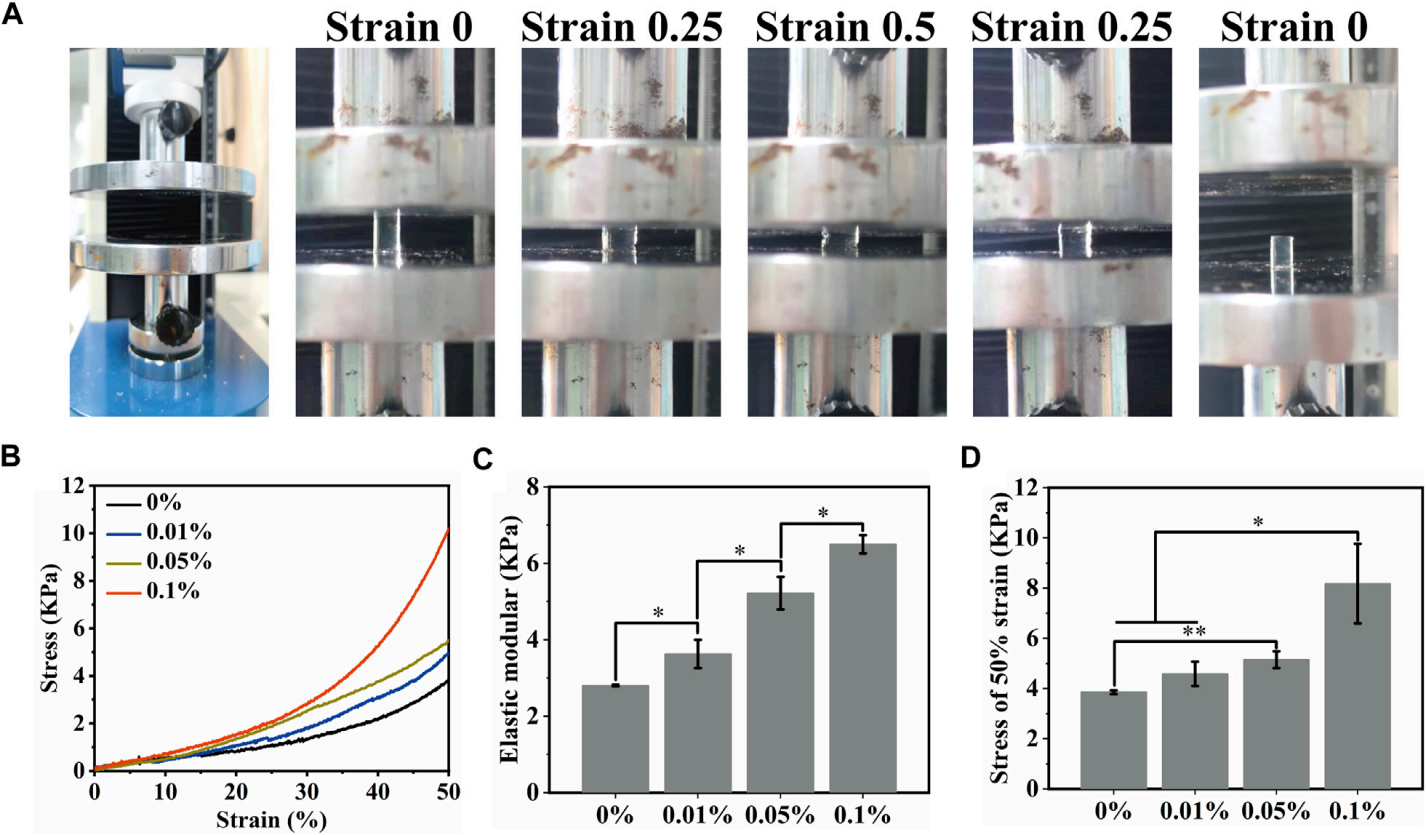
Mechanical properties of MeCFO/GelMA hydrogels: **(A)** Hydrogel compression experiment; **(B)** Stress-strain curves of magnetic MeCFO-GelMA hydrogels; **(C)** Elastic modulus of MeCFO/GelMA hydrogels; **(D)** ultimate stress (50% stain) of MeCFO/GelMA hydrogels.

Osteosarcoma resection often results in bone defects of varying sizes. This means that MeCFO/GelMA hydrogels need some mechanical support. Hydrogels are flexible and can resist bending and squeezing to a certain extent. The images of the mechanical experiment were shown in Figure 5A. The mechanical properties of MeCFO/GelMA hydrogels were changed with the addition of MeCFO (Figure 5B). With the increase of MeCFO content, the mechanical properties of MeCFO/GelMA hydrogel were also gradually enhanced, which revealed that magnetic nanoparticles improved the mechanical properties of hydrogels. It is due to the presence of nanoparticles that can arrange the orientation of polymer chains, leading to the formation of more regular structures and electrostatic interactions between nanoparticles and polymer functional groups. This resulted in increased cross-linking density and thus improved mechanical properties (Farzaneh et al., 2021). Figure 5C showed that MeCFO/GelMA hydrogels exhibited tunable compressive modulus in the range of 2.7–6.5 kPa. For hydrogels containing MeCFO, this upward trend was clear and statistically significant. The maximum value was also recorded in the composite containing 0.1% MeCFO, which was significantly higher than that of the blank group. The stress of 0%, 0.01%, 0.05%, and 0.1% MeCFO/GelMA hydrogels under 50% strain is 3.85 ± 0.07, 4.58 ± 0.48, 5.14 ± 0.33 and 8.18 ± 1.59 kPa, respectively (Figure 5D). Therefore, 0.1% MeCFO/GelMA hydrogel demonstrated better resistance to fracture at 50% strain, which possessed considerable hydrogel stiffness.

### 3.4 Rheological properties of MeCFO/GelMA hydrogel

**FIGURE 6:**
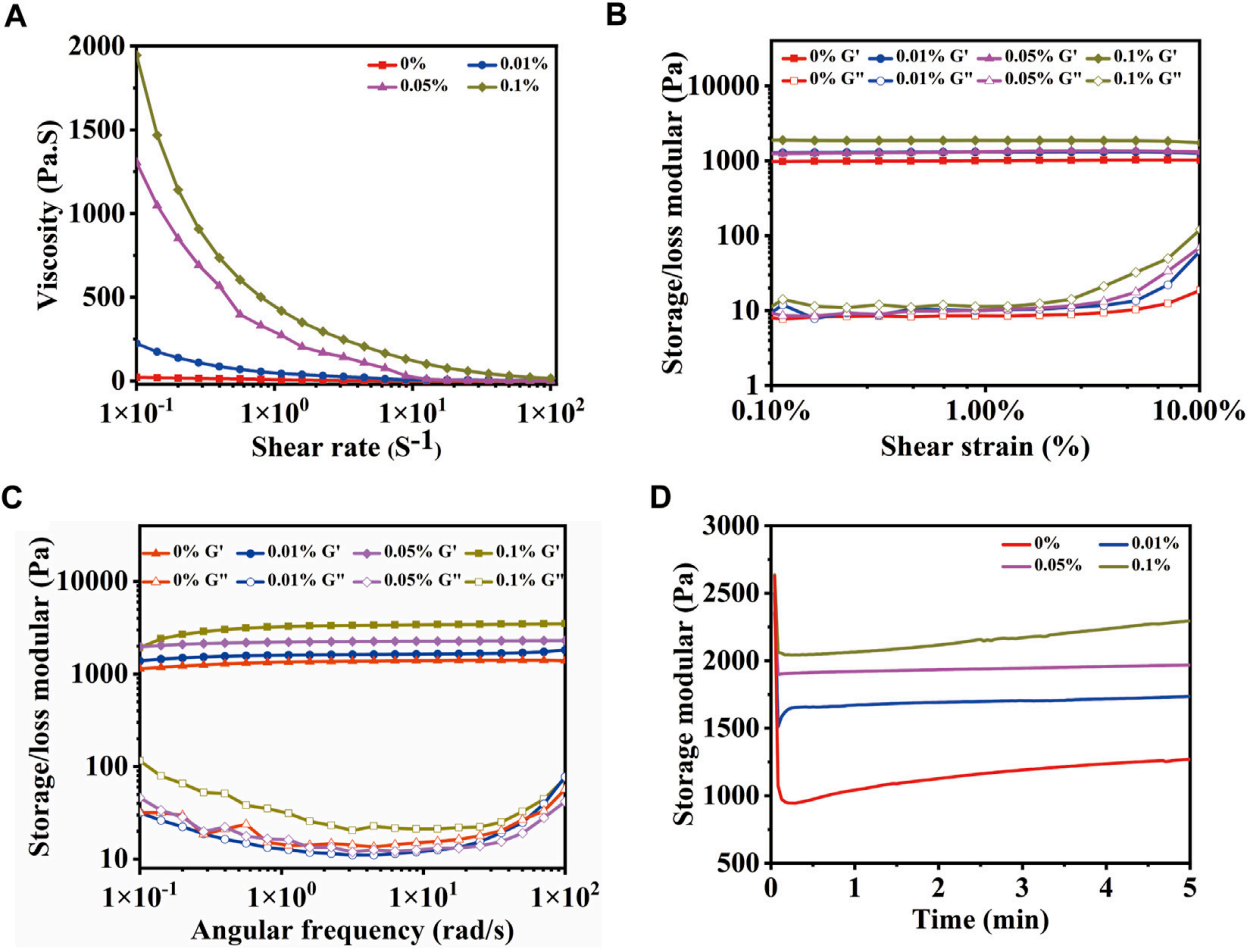
Rheological properties of MeCFO/GelMA hydrogels with 0%, 0.01%, 0.05%, and 0.1% CFO particles: **(A)** flow sweep scanning; **(B)** amplitude scanning; **(C)** frequency scanning; **(D)** time scanning.

As a temporary substrate for bone repair, the shear force of the hydrogel is also important in addition to the compression force, while new bone is formed. In Figure 6A, the viscosity of hydrogels varies with the shear rate. The relationship between shear stress and shear rate is nonlinear, which is manifested as shear thinning. The viscosity of MeCFO/GelMA gels at the low shear rate increased with the increase in the MeCFO ratio, which means that shear thinning performance was enhanced. This simulated the shear process of the hydrogel extruded from the printing cylinder (Meng et al., 2020). So MeCFO/GelMA hydrogel system can be extruded for 3D printing, which is beneficial for designing scaffolds for bone defects with different shapes. For the amplitude scanning (Figure 6B) and frequency scanning (Figure 6C), the energy storage modulus (G′) of hydrogels remained stable with the increase of shear strain or frequency, but their loss modulus (G″) increased when the shear strain was higher than 2% and the frequency was higher than 20 rad/s. With the increase of MeCFO content, the G′ and G″ was increased. It is because the nanoparticles act as reinforcers, improving the viscoelasticity of the hydrogel (Kuang et al., 2019). This result is consistent with the compression performance mentioned above. The results of time scanning measurements are shown in Figure 6D. The variation curve of the G′ of hydrogel with time is roughly stable, which indicates that hydrogel has sufficient mechanical properties and strong stability. With the increase of MeCFO content, the G′ of hydrogel was also enhanced. This stability over time is one of the desirable properties of extrudable materials (injectable materials, 3D printable materials, and so on) in filling defect sites (Arun Kumar et al., 2016). Rheological results show that MeCFO can significantly enhance G′ and G''. In particular, 0.1% MeCFO/GelMA hydrogel showed a wide linear viscoelastic region and good shear resistance, indicating satisfactory toughness.

### 3.5 Porosity and swelling studies of MeCFO/GelMA hydrogel

**FIGURE 7:**
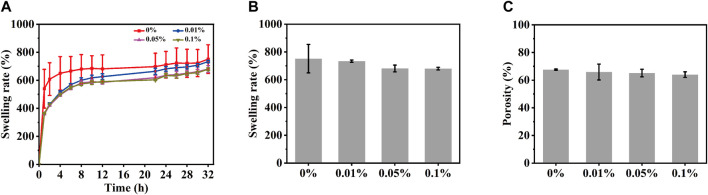
Porosity and swelling studies of MeCFO/GelMA hydrogels. **(A)** swelling rate; **(B)** swelling rate of equilibrium swelling; **(C)** porosity.

One of the most important properties of hydrogel materials is their ability to absorb water. The swelling properties of scaffolds used in tissue engineering are essential for cell attachment. For this purpose, the swelling behavior of magnetic hydrogels with different content of MeCFO was studied, which also indirectly indicated porosity (Ganji et al., 2010). The swelling state of all samples can be divided into two stages. The first stage occurs within 2 h and increases rapidly, while the second stage is a high plateau after 24 h, as shown in Figure 7A. All GelMA hydrogels swelled fastest within 2 h, then it starts to stabilize. After 24 h, the equilibrium state of swelling is reached. The equilibrium swelling rate (Figure 7B **)** of 0%, 0.01%, 0.05%, and 0.1% MeCFO/GelMA hydrogels are 751.7% ± 102.4%, 733.7% ± 8.2%, 680.9% ± 24.4%, and 679.6% ± 9.6%, respectively. In particular, GelMA hydrogel has the fastest swelling speed and the highest equilibrium swelling rate. As the number of nanoparticles in the hydrogel increased, the number of pores increased, but the size of the pores and their interconnections decreased (Bonhome-Espinosa et al., 2017). Therefore, the water absorption ability of the hydrogel decreased with the increase of nanoparticles. In addition to water absorption capacity and 3D-connected porous morphology, bone defect repair scaffolds should also have effective porosity, which should match the porosity range of natural bone (40%–65%) (Saravanan et al., 2017). The porosity of MeCFO/GelMA hydrogel was analyzed by the ethanol replacement method (Zhang et al., 2013). Figure 7C shows that the porosity of MeCFO/GelMA hydrogels is more than 60%. There was no statistically significant difference between the groups. It meets the porosity requirement of bone tissue.

### 3D printability of MeCFO/GelMA hydrogels and their structures

**FIGURE 8:**
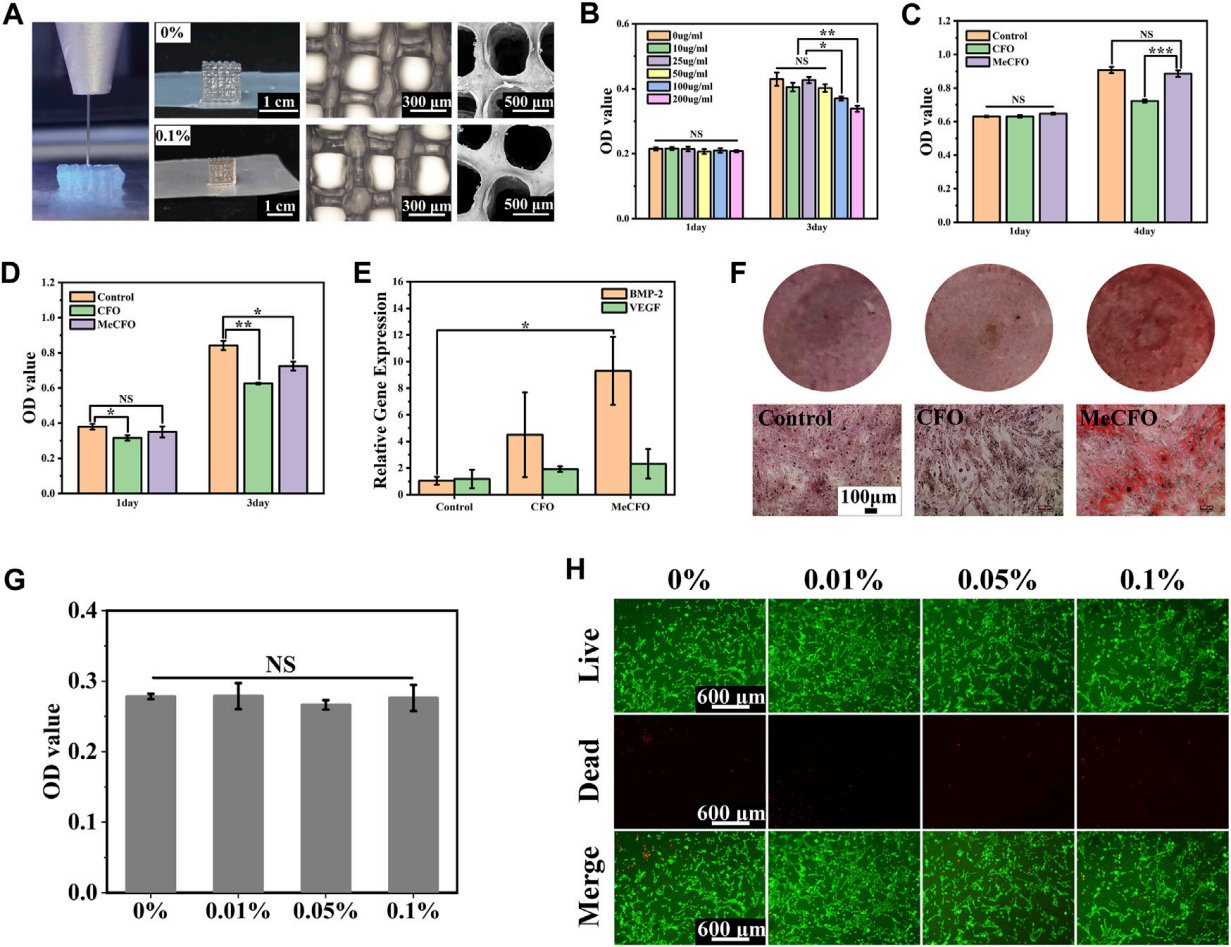
3D printing and cell experiment of MeCFO/GelMA hydrogels: **(A)** the morphology of 3D printed hydrogels; **(B)** the cytotoxicity (BMSCs) of MeCFO at different concentrations; **(C)** the cytotoxicity (BMSCs) comparison between CFO and MeCFO; **(D)** the cytotoxic effect of MeCFO on Saos-2 cells; **(E)** expression of osteogenic genes in MeCFO cultured with BMSCs; **(F)** the diagrams of alizarin red staining (BMSCs); **(G)** the cytocompatibility (BMSCs) of GelMA hydrogels with different amounts of MeCFO; **(H)** images of the live and dead cell staining of BMSCs on MeCFO/GelMA hydrogels.

The prepared MeCFO/GelMA hydrogel can be extruded into self-supporting uniform filaments by the virtue of the rheological properties (Figure 1C and Figure 8A). Figure 8A also showed the macro and micro morphology of 3D-printed MeCFO/GelMA hydrogel. The results showed that 3D printed hydrogels had a complete shape without distortion, deformation, and collapse. The pore size of the 3D-printed hydrogel was uniform. In addition, the hydrogel grid structure was visible and the diameter of the fibers looks alike. There is some distance between the layers, which conformed to the imported 3D scaffold model. This can provide a good three-dimensional culture environment for the cells (Yuan et al., 2018). Meanwhile, the performance of MeCFO/GelMA hydrogel can meet the requirements of 3D printing.

### 3.7 Cell experiments

In this study, we first conducted cytotoxicity experiments with different concentrations of MeCFO extracts to screen out the gradient of better cytocompatibility. Figure 8B showed no cytotoxicity at MeCFO concentrations less than 50 μg/mL. The concentration was consistent with 0.1% MeCFO content in GelMA hydrogel. In recent years, a large number of cell experiments have been conducted to evaluate the cytotoxicity of CFO nanoparticles *in vitro* (Abudayyak et al., 2017; Ahmad and Zhou, 2017; Panda et al., 2022). It has been reported that CFO nanoparticles with surface modification have lower cytotoxicity (Mushtaq et al., 2017). The cytocompatibility of MeCFO hydrogel on BMSCs was studied by the CCK-8 method. Compared with 50 μg/mL MeCFO, CFO at the same concentration showed significant cytotoxicity (Figure 8C). This indicates that methacrylate groups can improve the cytocompatibility of CFO. Saos-2 cells were selected to verify the antitumor effect of MeCFO. The CCK-8 results showed that 50 μg/mL MeCFO significantly reduced the activity of tumor cells (Figure 8D). In addition to anti-tumor through magnetothermal effects, MeCFO also had direct cytotoxicity to tumor cells. This may be related to tumor cells uptaking more nanoparticles, which was conducive to killing tumor cells targetable (Prijic et al., 2010; Wang and Thanou, 2010). Hence, 50 μg/mL MeCFO nanoparticles have good cytocompatibility with normal cells but have obvious cytotoxicity to tumor cells. Moreover, Fe^3+^ and Co^2+^ have a certain osteogenic effect (Glenske et al., 2018). It has previously been shown that the differentiation of BMSCs affected bone formation (Ghosh et al., 2019). RT-PCR results in this study showed that the expression of the osteogenic differentiation gene of BMP-2 in the 50 μg/mL MeCFO group was significantly increased compared with the control group (Figure 8E). Although there was no statistical difference in VEGF gene expression, there was also an upward trend in the MeCFO group when compared with CFO and the control group. In addition, osteoblasts produce extracellular calcium-deposited minerals to form bone tissue. Alizarin red staining was used to detect the amount of mineralization caused by the formation of bone nodules. Alizarin red staining results showed that the MeCFO group had the deepest staining color among the three experimental groups (Figure 8F). This suggests that 50 μg/mL MeCFO is beneficial to bone regeneration through bone mineralization.

For MeCFO/GelMA hydrogels, the cytotoxicity assay at day 4 in Figure 8G showed that all hydrogels had good cytocompatibility when cultured with BMSCs and there was no significant statistical difference between groups. It has been reported in the literature that the cytotoxicity of CFO is displayed in a dose- and size-dependent manner (Ansari et al., 2018). The higher concentration and smaller the size of CFO, the more cytotoxic it is. In this study, the final concentration of MeCFO was less than 0.1% and the particle size was large in the range of 20–100 nm. So the hydrogel had good cytocompatibility, which is similar to the conclusion of related literature (Ansari et al., 2016). The staining results of living cells and dead cells were consistent with the results of the cytotoxicity assay. Live and dead cell experiments showed that the cells grew well within 4 days after incubation with MeCFO/GelMA hydrogels. Most of the cells showed living cells with green fluorescence, while dead cells with red fluorescence were almost absent (Figure 8H). And the cells could be seen on the hydrogel pseudopodia and spread out well. These results suggest that MeCFO/GelMA hydrogels function as effective matrix supporting cell adhesion and growth. The introduction of MeCFO nanoparticles in small quantities does not affect the cytocompatibility of hydrogels. Considering the osteogenic differentiation ability of low content of MeCFO, 0.1% MeCFO-incorporated GelMA hydrogels will be promising for the bone regeneration.

## 4 Conclusion

Magnetic hyperthermia is a novel therapeutic strategy for osteosarcoma. In this work, a high-performance GelMA hydrogel loading with magnetic cobalt ferrite nanoparticles was designed for the magnetic hyperthermia treatment of osteosarcoma. First, the surface of CFO was chemically grafted with the methacrylate group. According to the results of features analysis such as TEM, EDX, FTIR, XPS, TG, and VSM, MeCFO not only retains good magnetic properties but also reduces agglomeration compared with CFO. Theoretical simulations showed that MeCFO could be used to raise temperatures to 40°C–45°C with magnetic fields, which is beneficial for magnetic hyperthermia. In addition, methacrylate groups can improve the cytocompatibility of CFO. Next, different solid contents of MeCFO were added to the GelMA solution and then MeCFO/GelMA hydrogel was prepared by UV light crosslinking. FTIR analysis confirmed the successful incorporation of CFO into MeCFO/GelMA hydrogel by the appearance of MeCFO functional groups. SEM image showed the hydrogel had an obvious porous and channel structure, which facilitates cell migration and nutrient exchange. So it promotes bone reconstruction after osteosarcoma resection. As the proportion of MeCFO increases, the pore size of MeCFO/GelMA hydrogels shrinks and the mechanical properties of hydrogels gradually strengthened. And then, the results of rheological properties are consistent with those of mechanical properties. In addition, MeCFO/GelMA hydrogels have good porosity and swelling rate. There was no statistical difference in their porosity and swelling rates. Moreover, we have demonstrated magnetic hydrogels were successfully 3D printed in a good shape. This shows that they are good magnetic bioprinting inks. Meanwhile, the cell experiment proved 50 μg/mL MeCFO can not only kill Saos-2 cells but also stimulate the osteogenic differentiation and mineralization of BMSCs. The results of CCK-8 and staining of live and dead cells also proved the good biocompatibility of the magnetic hydrogels. Therefore, MeCFO/GelMA hydrogels have great potential in the treatment of osteosarcoma.

## Data Availability

The original contributions presented in the study are included in the article/Supplementary Material, further inquiries can be directed to the corresponding authors.
